# The Significant Associations between Epigenetic Clocks and Bladder Cancer Risks

**DOI:** 10.3390/cancers16132357

**Published:** 2024-06-27

**Authors:** Yang Deng, Chia-Wen Tsai, Wen-Shin Chang, Yifan Xu, Maosheng Huang, Da-Tian Bau, Jian Gu

**Affiliations:** 1 Department of General Surgery, Ruijin Hospital, Shanghai Jiao Tong University School of Medicine, Shanghai 200031, China; 2 Department of Epidemiology, The University of Texas MD Anderson Cancer Center, Houston, TX 77030, USA; 3 Terry Fox Cancer Research Laboratory, Department of Medical Research, China Medical University Hospital, Taichung 404327, Taiwan; 4 Department of Bioinformatics and Medical Engineering, Asia University, Taichung 41354, Taiwan

**Keywords:** bladder cancer, biologic age, DNA methylation, SNP, epigenetic clock, Mendelian randomization

## Abstract

**Simple Summary:**

Bladder cancer, an age-related disorder, primarily affects individuals aged 65 and older. Accelerated biological aging, which is linked to increased cancer susceptibility, raises questions about its association with bladder cancer risk. We evaluated four widely recognized epigenetic clocks—HannumAge, HorvathAge, GrimAge, and PhenoAge—for their relationship with bladder cancer risk in a large case–control study. By leveraging genome-wide-association study (GWAS) data and constructing weighted genetic risk scores (GRS), we found that higher HannumAge and HorvathAge GRS were significantly associated with increased bladder cancer risk (OR = 1.69, 95% CI: 1.44–1.98, *p* = 1.56 × 10^−10^ and OR = 1.09, 95% CI: 1.00–1.19, *p* = 0.04, respectively). Mendelian randomization (MR) analysis using inverse-variance weighting (IVW) confirmed these associations, which was also supported by sensitivity analyses. However, GrimAge and PhenoAge showed no significant association with bladder cancer risks. Our findings highlighted the link between accelerated biological aging and elevated bladder cancer risk.

**Abstract:**

Bladder cancer is an age-related disease, with over three-quarters of cases occurring in individuals aged 65 years and older. Accelerated biological aging has been linked to elevated cancer risks. Epigenetic clocks serve as excellent predictors of biological age, yet it remains unclear whether they are associated with bladder cancer risk. In this large case–control study, we assessed the associations between four well-established epigenetic clocks—HannumAge, HorvathAge, GrimAge, and PhenoAge—and bladder cancer risk. Utilizing single nucleotide polymorphisms (SNPs), which were identified in a genome-wide association study (GWAS), linked to these clocks as instruments, we constructed a weighted genetic risk score (GRS) for each clock. We discovered that higher HannumAge and HorvathAge GRS were significantly associated with increased bladder cancer risk (OR = 1.69 per SD increase, 95% CI, 1.44–1.98, *p* = 1.56 × 10^−10^ and OR = 1.09 per SD increase, 95% CI, 1.00–1.19, *p* = 0.04, respectively). Employing a summary statistics-based Mendelian randomization (MR) method, inverse-variance weighting (IVW), we found consistent risk estimates for bladder cancer with both HannumAge and HorvathAge. Sensitivity analyses using weighted median analysis and MR-Egger regression further supported the validity of the IVW method. However, GrimAge and PhenoAge were not associated with bladder cancer risk. In conclusion, our data provide the first evidence that accelerated biological aging is associated with elevated bladder cancer risk.

## 1. Introduction

Bladder cancer ranks as the fourth most prevalent cancer among men and the sixth most commonly diagnosed cancer overall in the United States [1]. Smoking and occupational exposure are two major modifiable risk factors for bladder cancer [2,3,4,5,6]. Genetic susceptibility and gene–environment interactions play important roles in bladder cancer etiology [7,8,9,10]. Bladder cancer, like most other cancers, is an age-related disease [11]. The age-adjusted incidence rate in elderly persons (65 years and older) is 5-fold higher than that in middle-aged persons (50–64 years), at 106 per 100,000 compared to 20.8 per 100,000. For those younger than 50 years, the rate is significantly lower at 1.1 per 100,000, which is 100 times lower than that for the elderly. Three-quarters of bladder cancer patients are over 65 years old, with an average age of diagnosis of 73 [12]. The increased cancer risk associated with advancing age is a result of multiple factors, including, but not limited to, the accumulating somatic mutations due to less efficient DNA repair mechanisms, increased exposure to tobacco and carcinogens, and a weakened immune system [13].

While chronological age remains the primary risk factor for most cancers, including bladder cancer, the individual risks among the elderly at the same chronological age vary considerably. Conversely, biological age, which incorporates data from clinical and/or biological markers linked to aging, provides a more accurate reflection of an individual’s physiology and susceptibility to age-related diseases. Biological age correlates with chronological age but provides additional information in risk assessments for age-related conditions beyond chronological age [14,15]. In recent years, various biological age predictors have been proposed and validated, including clinical based measures (e.g., blood chemistries, blood pressure, lung function, and frailty indices), telomere length, epigenetic clocks, “omics”-based predictors, and composite biomarker predictors [14,15,16]. Accelerated aging, i.e., individuals whose predicted biological age is greater than their chronological age, has been associated with increased risks of diseases and mortality [14,15,16,17,18,19]. Among all the currently available biological age predictors, epigenetic clocks are believed to be the most robust due to their high correlation with chronological age and the prediction of aging-related diseases and mortality [20,21,22].

Epigenetic alterations, notably DNA methylation, manifest across various cell types and tissues as age advances. Previous studies have identified numerous specific age-related CpG methylation sites and used the methylation levels of these CpG sites to develop algorithms to predict a person’s BA; these algorithms were termed “epigenetic clocks” [20,21,22]. The first-generation epigenetic clock, HannumAge, is based on 71 age-related CpG sites identified from leukocytes [23], and HorvathAge used 353 age-related CpG sites across a broad spectrum of human tissues and cell types [24]. Recently, several second-generation epigenetic clocks were developed using a more sophisticated scheme. PhenoAge used 513 CpGs associated with a “phenotypic age” estimator consisting of chronological age and nine clinical biomarkers [25], while GrimAge is a linear model that combines chronological age, sex, and 1030 CpGs linked to seven plasma proteins and smoking intensity (pack-years) [26].

Accumulating evidence from observational studies has suggested that these different epigenetic clocks are associated with overall and specific cancer risks, but the results are not consistent [18,19,26,27,28,29,30]. Given that DNA methylation is an intermediate biomarker influenced by environmental exposures, disease statuses, and various confounding factors, concerns often arise regarding reverse causation and assay accuracy in observational studies when evaluating epigenetic clocks as cancer risk factors. Mendelian randomization (MR) studies have increasingly been employed to discern causality between risk factors/biomarkers and cancer risk [31,32,33] since they are less prone to being affected by reverse causation and other concerns compared to traditional observational studies. Two recent studies have used the MR approach to assess the association of genetically predicted HannumAge, HorvathAge, PhenoAge, and GrimAge with the risks of breast, prostate, lung, colorectal, and ovarian cancers [34,35]. In this large case–control study, we used the MR approach to evaluate the associations of these four epigenetic clocks with bladder cancer risk.

## 2. Materials and Methods

### 2.1. Study Population

This is a case–control study, and the study details have been described previously [36,37,38,39]. All the cases had histologically confirmed urothelial cell carcinomas. They were recruited from the University of Texas MD Anderson Cancer Center (MDACC) and Baylor College of Medicine. Controls were recruited from a large physician group in the Houston metropolitan area, Kelsey-Seybold Clinics. Controls were matched to cases based on age (±1 years) and gender. All the study participants were European descendants. All participants signed informed consent. This study was approved by the institutional review boards of MDACC, Baylor College of Medicine, and Kelsey-Seybold Clinics.

### 2.2. Genotyping and Imputation

The genotyping and imputation procedures have been described previously [36,37,38,39]. We used the Michigan Imputation Server (https://imputationserver.sph.umich.edu/, accessed on 2 January 2023) for imputation using the 1000 Genomes Project Phase 3 data as the reference panel. GWAS has identified 9 SNPs associated with HannumAge, 24 SNPs with HorvathAge, 11 SNPs with PhenoAge, and 4 SNPs with GrimAge [34,35]. These SNPs were used as genetic instruments for MR analyses.

### 2.3. Statistical Analysis and Mendelian Randomization Analysis

We used Student’s *t*-test to compare continuous variables (e.g., age and GRS) and chi-square test or Fisher’s exact test to compare the categorical variables (e.g., smoking status, dichotomized GRS, and genotype frequency) between cases and controls. We constructed a weighted GRS using SNPs for each of the four epigenetic clocks as we previously described [37,38,39,40]. We used multivariable linear regression to analyze the association of continuous GRS with bladder cancer risk adjusting for age and smoking status. We also analyzed GRS as a categorical variable by dichotomizing or quadrisecting individuals based on the median or quartile distribution of GRS in controls. We used multivariable logistic regression to analyze categorical GRS and bladder cancer risk adjusting for age and smoking status. We used the inverse-variance weighting (IVW) method for MR analysis [37,38,39], and MR-Egger [41] and weighted median tests [42] for sensitivity analyses. All the statistical analyses and MR analyses were done using R software (version 4.3.2) [43]. All *p*-values were two-sided, with a significance level of 0.05.

## 3. Results

### 3.1. Selected Characteristics of Study Population

This study consists of 2030 pairs of bladder cancer cases and heathy controls (Table 1). The controls were matched 1:1 to the cases based on age (±1 year) and gender. The average age (with standard deviation) at diagnosis for cases was 64.70 (10.64) years, and for controls at recruitment, it was 64.59 (10.22) years. Nearly 80% of the cases were male, reflecting the male dominance of the incidence of bladder cancer. The cases had significantly more former and current smokers (51.38% and 19.99%, respectively) than the controls (43.63% and 12.65%, respectively) (*p* < 0.001), reaffirming that smoking is a significant risk factor for bladder cancer.

### 3.2. Association of Individual Epigenetic Clock Instrument SNPs with Bladder Cancer Risk

There were a total of 48 GWAS-identified SNPs associated with different epigenetic clocks, including 9 SNPs with HannumAge, 24 SNPs with HorvathAge, 11 SNPs with PhenoAge, and 4 SNPs with GrimAge. Only seven SNPs were significantly associated with bladder cancer risk at *p* < 0.05 (Table 2). Notably, among the nine HannumAge SNPs, four reached nominal significance (*p* < 0.05) and two remained significant after Bonferroni correction (*p* < 0.05/48 = 1.0 × 10^−3^). These two highly significant SNPs were rs1005277 near the ZNF25 gene (OR = 1.45, 95% CI, 1.29–1.62, *p* = 3.68 × 10^−10^) and rs12417758 in a long non-coding RNA (lncRNA) gene, RP11-867G23.13 (OR = 1.22, 95% CI, 1.11–1.34, *p* = 3.66 × 10^−5^). One HorvathAge SNP and two PhenoAge SNPs also reached *p* < 0.05; however, the only significant HorvathAge SNP (rs4240228) is in complete linkage disequilibrium (LD) with one of the significant HannumAge SNPs (rs4383328), and the top significant PhenoAge SNP, rs6531114, is also in high LD with rs4383328 (D’ = 0.84, R^2^ = 0.69).

### 3.3. Genetic Risk Score (GRS)

**Figure 1:**
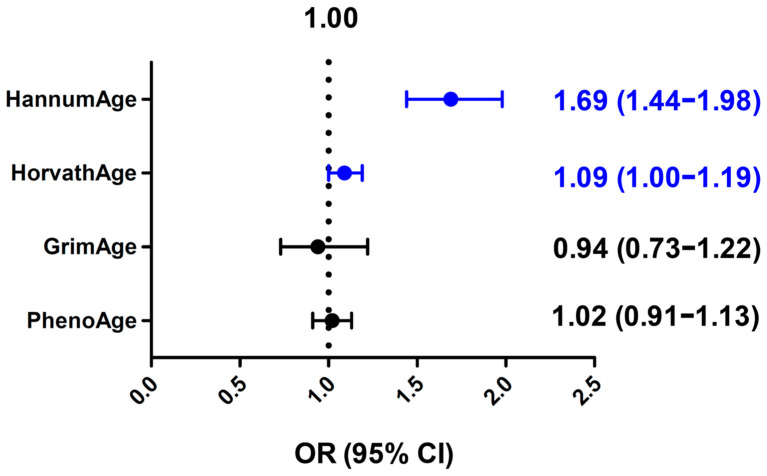
Associations of genetic risk score (GRS) of different epigenetic clocks with bladder cancer risks. Odds ratio (OR) per standard deviation increase and 95% confidence interval (CI) were calculated by multivariable linear regression analysis. Blue color indicates statistically significant.

We developed a weighted GRS for these four epigenetic clocks using the above SNPs as genetic instruments and compared the mean GRS between the cases and controls (Table 3). The GRS for HannumAge was significantly higher in the cases than the controls (mean ± SD: 2.33 ± 0.43 vs. 2.24 ± 0.39, *p* = 1.56 × 10^−10^), and the GRS for HorvathAge was slightly higher in the cases than the controls (7.83 ± 0.73 vs. 7.78 ± 0.77, *p* = 0.04). In multivariable linear regression analyses, an increasing GRS for HannumAge was strongly associated with an increased risk of bladder cancer (OR = 1.69 per SD increase, 95% CI, 1.44–1.98), while an increasing GRS for HorvathAge was modestly associated with an increased risk of bladder cancer (OR = 1.09 per SD increase, 95% CI, 1.00–1.19) (Figure 1). The GRS for GrimAge or PhenoAge was not associated with altered bladder cancer risks (OR = 0.94, 95% CI, 0.73–1.22 and OR = 1.02, 95% CI, 0.91–1.13, respectively) (Figure 1).

We also analyzed GRS as a categorical variable by dichotomizing or quadrisecting individuals (Table 4). In multivariable logistic regression analyses, when dichotomized at the median GRS in controls, individuals with a high GRS for HannumAge exhibited a 1.31-fold (95% CI, 1.15–1.49) increased risk of bladder cancer compared to those with a low GRS (*p* = 5.89 × 10^−5^). In quartile analysis, those in the highest quartile GRS group had a 1.71-fold (95% CI, 1.42–2.06) increased risk of bladder cancer compared to those in the lowest quartile group (*p* = 1.31 × 10^−8^). Modestly increased bladder cancer risks were observed for individuals with high GRS for HorvathAge compared to those with low GRS in the dichotomized and quartile analyses, but these analyses did not reach statistical significance (Table 4).

### 3.4. Summary-Statistics-Based Mendelian Randomization Analyses

**Figure 2:**
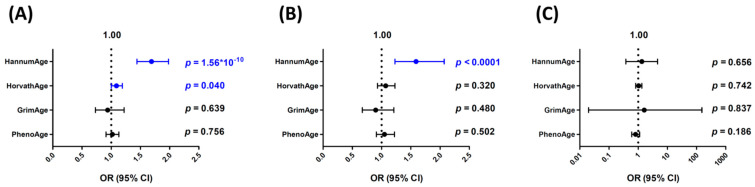
Summary-statistics-based Mendelian randomization (MR) analyses for the associations of different epigenetic clocks with bladder cancer risks. Odds ratio (OR) per standard deviation increase and 95% confidence interval (CI) were calculated using the (**A**) inverse-variance weighting (IVW), (**B**) weighted median, and (**C**) MR–Egger regression methods. Blue color indicates statistically significant.

We then performed a summary-statistics-based MR analysis, IVW, and weighted median and MR–Egger regression for sensitivity analyses (Figure 2). Consistent with the GRS analyses, HannumAge was strongly associated with an increased risk of bladder cancer (OR = 1.64 per SD increase, 95% CI, 1.41–1.92, *p* < 0.0001), and HorvathAge was also associated with an increased risk of bladder cancer (OR = 1.10 per SD increase, 95% CI, 1.01–1.20, *p* = 0.037). GrimAge or PhenoAge were not associated with altered bladder cancer risks in the IVW analysis.

In sensitivity analyses, the weighted median analysis gave similar risk estimates for all four epigenetic clocks versus the IVW method. The ORs (95% CI) per SD increase for HannumAge, HorvathAge, GrimAge, and PhenoAge were 1.59 (1.23–2.07), 1.07 (0.93–1.23), 0.90 (0.67–1.21), and 1.05 (0.91–1.22), respectively. MR–Egger regression analysis showed a similar trend for HannumAge and HorvathAge (OR = 1.33, 95% CI, 0.38–4.63 and OR = 1.04, 95% CI, 0.82–1.33, respectively), although it did not reach statistical significance (Figure 2). The MR–Egger intercepts were not significantly deviated from zero (intercept = 0.055, 95% CI, −0.259–0.369, *p* = 0.731 and 0.016, 95% CI, −0.050–0.081, *p* = 0.641, respectively, for HannumAge and HorvathAge), indicating no evidence of directional pleiotropy.

## 4. Discussion

This is the first epidemiological study to evaluate the associations of epigenetic clocks and bladder cancer risks. Using both an individual data-based GRS analysis and summary-statistics-based MR approaches, we observed strong positive associations between higher HannumAge acceleration and an increased bladder cancer risk and a modest positive association between higher HorvathAge and an increased bladder cancer risk, whereas GrimAge or PhenoAge were not associated with bladder risks.

Given that aging is a major risk factor for bladder cancer, individuals who have an accelerated biological age are believed to have higher risks of bladder cancer. Several measures of biological age have been developed, including clinical measures, telomere length, epigenetic clocks, “omics”-based predictors, and composite biomarkers [14,15,16], and have been used to assess their associations with cancer risks [17,18,19]. Epigenetic clocks are arguably the best predictor of biological age, and increasing epidemiological studies have suggested that epigenic clocks are associated with overall and specific cancer risks, although the results are not entirely consistent [18,19,20,21,22,26,27,28,29,30].

Recently, MR studies using genetic instruments as a proxy have been widely used to infer the causality between risk factors/biomarkers and cancer risks [31,32,33]. MR study is less likely to be affected by reverse causation and assay reproducibility compared to observational studies. Two recent MR studies have evaluated the association of genetically predicted HannumAge, HorvathAge, PhenoAge and GrimAge with the risks of breast, prostate, lung, colorectal, and ovarian cancers [34,35]. Higher GrimAge acceleration, but not the other three epigenetic clocks, was found to increase the risk of colorectal cancer. In addition, there was suggestive evidence for the association between GrimAge and prostate cancer and between HorvathAge and lung cancer [35]. It is apparent that the associations between epigenetic clocks and cancer risks are not only cancer-type-dependent, but also clock-type-specific. In our current study, we found that the two first-generation epigenetic clocks, HannumAge and HorvathAge, but not the two second-generation epigenetic clocks, were associated with bladder cancer risks. It is unlikely that this is due to horizontal pleiotropy, as the MR–Egger intercept did not significantly deviate from zero. Different epigenetic clocks were trained on different outcomes, tissues, and populations, and they may capture distinct aging pathways, processes, and mechanisms, leading to the differential associations with aging outcomes including cancer risks [44]. HannumAge and HorvathAge were trained using chronological age as the sole outcome and are the most robust predictors of chronological age. The HannumAge was trained on DNA methylation data from whole blood [23], and the HorvathAge clock is a multi-tissue predictor trained on methylation levels from 51 healthy tissues and cell types [24]. Their associations with bladder cancer risk are not surprising since they are strongly correlated with chronological age. On the other hand, the second-generation epigenetic clocks were developed using a more sophisticated scheme. PhenoAge used 513 CpGs associated with a “phenotypic age” outcome consisting of chronological age and nine clinical biomarkers (albumin, creatinine, serum glucose, C-reactive protein, lymphocyte percentage, mean cell volume, red cell distribution width, alkaline phosphatase, and white blood cell count) [25], while GrimAge is a linear combination of chronological age, sex, and 1030 CpG sites associated with seven plasma proteins (adrenomedullin, beta-2-microglobulim, cystatin C, GDF-15, leptin, PAI-1, and TIMP-1) and smoking pack-years [26]. These second-generation clocks capture complex biological processes related to aging. They may serve as excellent indicators of mortality, yet their associations with cancer risks may prove intricate and diverse. Further studies are needed to determine the associations of these various epigenetic clocks with different cancer types.

Because of the distinct training processes of these epigenetic clocks, there are minimal overlaps among the CpG sites included in each epigenetic clock. One of the rare, overlapping loci among three clocks maps to an antisense RNA (AC104623.2) on chromosome 2p24.2 and is significantly associated with bladder cancer risk in our study. The biological function of AC104623.2 is unknown. Surprisingly, four out of nine SNPs in the HannumAge clock were significantly associated with bladder cancer risks (*p* < 0.05), and two remained significant after Bonferroni correction, while the other three clocks only had one additional independent SNP (rs752223 in PhenoAge) reaching nominal significance. The most significant SNP associated with bladder cancer is rs1005277. A previous meta-analysis of GWAS identified this SNP as the most significant SNP associated with HannumAge, the only one reaching genome-wide significance [45]. This SNP is located on chromosome 10p11.21, and its nearest gene is ZNF25. ZNF25 is a zinc finger protein containing a KRAB transcriptional repressor domain. The knockdown of ZNF25 resulted in the striking upregulation of MMP1, RANBP3L, and LGR5 expression during the osteoblast differentiation of human skeletal stem cells [46]. There has been no study investigating the molecular mechanisms of ZNF25 in the aging process or bladder carcinogenesis. The second highly significant SNP associated with bladder cancer is rs12417758, mapping to a lncRNA (RP11-867G23.13) on chromosome 11q13.1. The function of RP11-867G23.13 is unknown, but DNA methylation and SNPs at RP11-867G23.13 have been associated with increased risks of breast cancer in a recent study [47]. Further functional studies are needed to investigate the molecular mechanisms underlying the associations of these significant SNPs and genes with the development of bladder cancer.

There are a few limitations to this study. First, the numbers of SNPs included in the epigenetic clocks are limited, and these SNPs only explain small proportions of the variability in epigenetic aging. Additional SNPs are needed to construct more robust genetic instruments to estimate epigenetic clocks more accurately. Second, although the sample size is large enough for our main analyses, the MR–Egger sensitivity analysis had relatively low power, leading to imprecise estimates with large confidence intervals. Third, we matched the cases and controls on chronological age and sex, and we adjusted for age, sex, and smoking status in the multivariable regression analyses, which may not fully account for chronological age, smoking, and other residual confounding effects. Finally, we only included European Americans in this study. The associations of epigenetic clocks with bladder cancer in other ethnicities warrant further study.

## 5. Conclusions

In conclusion, in this large case–control study, we found for the first time that accelerated biologic aging, as determined by the genetically predicted HannumAge and HorvathAge epigenetic clocks, is associated with increased bladder cancer risks. Future prospective observational studies and MR analyses are needed to confirm findings in different populations.

## Figures and Tables

**Table 1:** Selected characteristics of the study population.

Variables	Cases, N (%)	Controls, N (%)	*p*-Value
**Age, mean (SD)**	64.70 (11.11)	64.59 (10.22)	0.738
**Sex**			
**Male**	1606 (79.11)	1606 (79.11)	
**Female**	424 (20.89)	424 (20.89)	1
**Smoking Status**			
**Never**	487 (28.63)	885 (43.72)	
**Former**	874 (51.38)	883 (43.63)	
**Current**	340 (19.99)	256 (12.65)	<0.001

**Table 2:** Individual associations of each epigenetic clock-related SNP with bladder cancer risk.

SNP ID	Chr.	Position	Gene	Allele	β	EAF *		OR (95% CI) **	*p*-Value
						Cases	Controls		
**HannumAge**									
rs1005277 ***	**10**	**38218259**	**ZNF25**	**A/C**	**0.301**	**0.320**	**0.246**	**1.45 (1.29–1.62)**	**3.68 × 10^−10^**
**rs12417758**	**11**	**66076360**	**RP11-867G23.13**	**C/T**	**0.209**	**0.463**	**0.416**	**1.22 (1.11–1.34)**	**3.66 × 10^−5^**
**rs4383328**	**2**	**16693124**	**AC104623.2**	**T/A**	**0.189**	**0.742**	**0.716**	**1.13 (1.02–1.25)**	**0.019**
**rs1598856**	**4**	**103446115**	**NFKB1**	**A/G**	**0.186**	**0.478**	**0.449**	**1.11 (1.01–1.22)**	**0.031**
rs34970912	16	73068163	ZFHX3	G/C	0.521	0.022	0.017	1.29 (0.92–1.81)	0.144
rs10786282	10	98122808	OPALIN	G/A	0.360	0.795	0.781	1.07 (0.96–1.2)	0.231
rs4838595	10	49675247	ARHGAP22	C/T	0.258	0.873	0.872	1.03 (0.9–1.18)	0.671
rs3093956	6	31426967	-	C/T	0.243	0.154	0.156	0.98 (0.86–1.11)	0.746
rs111731678	7	130418744	KLF14	T/A	0.227	0.821	0.815	1 (0.88–1.14)	0.967
**HorvathAge**									
**rs4240228**	**2**	**16688759**	**AC104623.2**	**T/G**	**0.255**	**0.742**	**0.716**	**1.13 (1.02–1.25)**	**0.019**
rs6577536	1	8910110	ENO1	A/G	0.197	0.494	0.471	1.1 (1–1.2)	0.054
rs10447389	6	25642577	ZFP57	G/A	0.276	0.727	0.714	1.08 (0.98–1.2)	0.124
rs1511762	18	42119324	LINC01478	T/C	0.264	0.229	0.217	1.09 (0.97–1.22)	0.133
rs2492286	3	128336298	RPN1	T/G	0.281	0.150	0.135	1.1 (0.96–1.27)	0.156
rs3917672	1	169592981	SELP	G/A	0.262	0.516	0.533	0.94 (0.85–1.03)	0.171
rs7550821	1	208029947	C1orf132	C/T	0.255	0.767	0.780	0.93 (0.83–1.03)	0.175
rs12043492	1	39457006	AKIRIN1	T/C	0.217	0.445	0.431	1.07 (0.97–1.17)	0.185
rs10732882	11	57111693	P2RX3	G/T	0.241	0.592	0.601	0.94 (0.86–1.04)	0.247
rs75243280	22	17601466	CECR6	C/T	0.232	0.273	0.266	1.07 (0.95–1.22)	0.273
rs144317085	4	105806108	RP11-556I14.2	A/T	0.514	0.972	0.971	1.15 (0.87–1.52)	0.324
rs2736099	5	1287340	TERT	A/G	0.233	0.346	0.329	1.06 (0.95–1.18)	0.333
rs1726672	1	236519502	EDARADD	C/T	0.204	0.679	0.687	0.96 (0.86–1.06)	0.371
rs7627756	3	160217483	KPNA4	A/G	0.216	0.558	0.554	1.03 (0.94–1.13)	0.474
rs12903325	15	50353277	ATP8B4	G/T	0.222	0.239	0.236	1.04 (0.93–1.16)	0.511
rs12666349	7	31728180	PPP1R17	T/C	0.255	0.853	0.851	0.96 (0.82–1.13)	0.624
rs6414374	3	150001224	LINC01214	A/G	0.321	0.146	0.139	1.03 (0.9–1.19)	0.631
rs10735418	12	107343376	RP11-412D9.4	T/C	0.195	0.625	0.625	1.02 (0.93–1.12)	0.69
rs10949481	6	18121029	NHLRC1	A/T	1.082	0.960	0.956	1.04 (0.83–1.31)	0.705
rs34003787	16	73071381	ZFHX3	T/C	0.324	0.064	0.067	0.97 (0.8–1.18)	0.766
rs79111787	3	47715545	SMARCC1	C/T	0.908	0.009	0.009	1.06 (0.67–1.7)	0.794
rs2275558	1	164529120	PBX1	G/A	0.234	0.913	0.901	0.97 (0.74–1.29)	0.855
rs1488106	3	168859006	MECOM	T/C	0.183	0.360	0.361	1 (0.91–1.1)	0.942
rs57941717	21	38374179	RIPPLY3	T/G	0.291	0.237	0.233	1 (0.9–1.12)	0.972
**GrimAge**									
rs887466	6	31143511	POU5F1	G/A	0.193	0.605	0.620	0.95 (0.87–1.05)	0.329
rs4065321	17	38143548	PSMD3	C/T	0.170	0.445	0.459	0.96 (0.87–1.05)	0.359
rs17094148	10	101280279	LINC01475	G/A	0.180	0.301	0.289	1.05 (0.94–1.16)	0.384
rs9386796	6	109618704	CCDC162P	T/C	0.198	0.459	0.451	1.01 (0.92–1.11)	0.873
**PhenoAge**									
**rs6531114**	**2**	**16617781**	**AC010880.1**	**C/T**	**0.254**	**0.766**	**0.743**	**1.13 (1.02–1.26)**	**0.023**
**rs752223**	**1**	**60433076**	**RN7SL475P**	**G/A**	**0.560**	**0.919**	**0.931**	**0.83 (0.69–0.99)**	**0.035**
rs11190127	10	101271982	LINC01475	A/C	0.248	0.378	0.367	1.04 (0.94–1.15)	0.447
rs116853700	17	55466295	MSI2	A/G	0.552	0.029	0.033	0.9 (0.69–1.17)	0.448
rs3829957	17	3378876	ASPA	C/T	0.380	0.806	0.801	1.04 (0.93–1.17)	0.456
rs678553	1	236525447	EDARADD	T/C	0.326	0.682	0.684	0.98 (0.88–1.09)	0.681
rs1142345	6	18130918	TPMT	T/C	0.823	0.961	0.957	1.04 (0.83–1.31)	0.716
rs7228835	18	41969071	LINC01478	G/C	0.514	0.893	0.890	1.03 (0.88–1.19)	0.739
rs11253338	10	759559	DIP2C	T/C	0.285	0.182	0.183	1.02 (0.9–1.15)	0.754
rs1990053	7	44925896	PURB	A/G	0.257	0.440	0.436	0.99 (0.91–1.09)	0.914
rs73028070	11	122681835	UBASH3B	G/A	0.433	0.944	0.944	0.99 (0.8–1.22)	0.931

* EAF: Effect allele frequency; ** Multivariable logistic regression analysis, adjusted by age and smoking status; *** SNPs significantly associated with bladder cancer risks are shown in bold.

**Table 3:** Comparisons of genetic risk score (GRS) between the cases and controls.

Epigenetic Clocks	Number of SNPs	GRS, Mean (SD)		*p*-Value
		Controls	Cases	
**HannumAge**	9	2.24 (0.39)	2.33 (0.43)	1.56 × 10^−10^
**HorvathAge**	24	7.78 (0.77)	7.83 (0.73)	0.040
**GrimAge**	4	0.68 (0.25)	0.68 (0.26)	0.639
**PhenoAge**	11	6.33 (0.62)	6.34 (0.61)	0.756

**Table 4:** Associations of categorical genetic risk score (GRS) of HannumAge and HorvathAge with bladder cancer risk.

GRS	Control, N (%)	Case, N (%)	OR * (95% CI)	*p*-Value
**HannumAge**				
**Dichotomize**				
Low	1015 (54.16)	859 (45.84)	1 (reference)	
High	1015 (46.43)	1171 (53.57)	1.31 (1.15–1.49)	5.89 × 10^−5^
**Quartile**				
1 (lowest)	508 (56.70)	388 (43.30)	1 (reference)	
2	507 (51.84)	471 (48.16)	1.22 (1.00–1.47)	0.045
3	508 (51.21)	484 (48.79)	1.19 (0.98–1.44)	0.075
4 (highest)	507 (42.46)	687 (57.54)	1.71 (1.42–2.06)	1.31 × 10^−8^
**HorvathAge**				
**Dichotomize**				
Low	1015 (51.55)	954 (48.45)	1 (reference)	
High	1015 (48.54)	1076 (51.46)	1.10 (0.97–1.26)	0.140
**Quartile**				
1 (lowest)	508 (52.32)	463 (47.68)	1 (reference)	
2	507 (50.80)	491 (49.20)	1.11 (0.92–1.33)	0.298
3	508 (48.61)	537 (51.39)	1.17 (0.97–1.40)	0.103
4 (highest)	507 (48.47)	539 (51.53)	1.15 (0.96–1.39)	0.129

* Multivariable logistic regression analysis, adjusted by age and smoking status.

## Data Availability

The data that support the findings of this study are available on request from the corresponding author. The data are not publicly available due to privacy or ethical restrictions.
